# Lipid Profiles After Changes in Alcohol Consumption Among Adults Undergoing Annual Checkups

**DOI:** 10.1001/jamanetworkopen.2025.0583

**Published:** 2025-03-12

**Authors:** Takahiro Suzuki, Sho Fukui, Tomohiro Shinozaki, Taku Asano, Toshiko Yoshida, Jiro Aoki, Atsushi Mizuno

**Affiliations:** 1 Department of Cardiovascular Medicine, St Luke’s International Hospital, Tokyo, Japan; 2 Department of Public Health, Institute of Science Tokyo, Tokyo, Japan; 3 Division of Rheumatology, Inflammation, and Immunity, Department of Medicine, Brigham and Women’s Hospital and Harvard Medical School, Massachusetts; 4 Immuno-Rheumatology Center, St Luke’s International Hospital, Tokyo, Japan; 5 Department of Emergency and General Medicine, Kyorin University School of Medicine, Tokyo, Japan; 6 Department of Information and Computer Technology, Faculty of Engineering, Tokyo University of Science, Tokyo, Japan; 7 Graduate School of Nursing Science, St Luke’s International University, Tokyo, Japan; 8 Tokyo Foundation for Policy Research, Tokyo, Japan

## Abstract

**Importance:**

Despite growing criticism of alcohol consumption due to its overall health risks, it remains unknown how changes in alcohol consumption, particularly cessation, affect lipid profiles outside of intense interventions.

**Objective:**

To clarify the association of alcohol initiation and cessation with subsequent changes in low-density lipoprotein cholesterol (LDL-C) and high-density lipoprotein cholesterol (HDL-C).

**Design, Setting, and Participants:**

This cohort study included individuals undergoing annual checkups at a center for preventive medicine in Tokyo, Japan, from October 2012 to October 2022. Individuals treated with lipid-lowering medications were excluded. Data were analyzed from May to December 2024.

**Exposures:**

Alcohol initiation (vs remaining abstainer) and cessation (vs continuing same alcohol intake) between 2 consecutive visits. One standard drink was equivalent to 10 g of pure ethanol.

**Main Outcomes and Measures:**

Change in LDL-C and HDL-C levels between 2 consecutive visits.

**Results:**

Among the 328 676 visits from 57 691 individuals (mean [SD] age, 46.8 [12.5] years; 30 576 female [53.0%]), the cohort for evaluating alcohol cessation comprised 49 898 visits among 25 144 participants (mean [SD] age, 49 [12.1] years; 12 334 female [49.1%]; mean [SD] LDL-C, 114.7 [28.4] mg/dL; mean [SD] HDL-C, 65.5 [16.4] mg/dL). Alcohol cessation was associated with changes in LDL-C of 1.10 mg/dL (95% CI, 0.76 to 1.45 mg/dL) among those discontinuing habits of fewer than 1.5 drinks/d, 3.71 mg/dL (95% CI, 2.71 to 4.71 mg/dL) for 1.5 to 3.0 drinks/d, and 6.53 mg/dL (95% CI, 5.14 to 7.91 mg/dL) for 3.0 or more drinks/d. Cessation was associated with a change in HDL-C of −1.25 mg/dL (95% CI, −1.41 to −1.09 mg/dL) among those discontinuing habits of fewer than 1.5 drinks/d, −3.35 mg/dL (−4.41 to −2.29 mg/dL) for 1.5 to 3.0 drinks/d, and −5.65 mg/dL (95% CI, −6.28 to −5.01 mg/dL) for 3.0 or more drinks/d. The cohort for evaluating alcohol initiation (107 880 visits; 29 042 participants) showed inverse dose-response associations.

**Conclusions and Relevance:**

In this cohort study of Japanese annual health checkup participants, alcohol initiation was associated with modest cholesterol improvement, whereas cessation was associated with less favorable changes. After alcohol reduction, lipid profile changes should be carefully monitored to optimize cardiovascular disease risk management at both individual and population levels.

## Key Points

**Question:**

Are changes in alcohol consumption, both initiation and cessation, associated with low-density lipoprotein cholesterol (LDL-C) and high-density lipoprotein cholesterol (HDL-C) in settings outside intense interventions?

**Findings:**

In this cohort study of 57 691 individuals undergoing annual health checkups at a center for preventive medicine in Japan, alcohol cessation was significantly associated with increased LDL-C and decreased HDL-C levels compared with continuing alcohol intake. Alcohol initiation showed opposite significant associations, with these changes more pronounced at higher consumption levels.

**Meaning:**

These results suggest that monitoring lipid profiles after changing alcohol habit is essential for optimizing cholesterol management.

## Teaser

This cohort study of Japanese adults receiving health checkups examines the association of starting or stopping drinking alcohol and changes in cholesterol levels.

## Introduction

Alcohol consumption has been identified as a major contributor to the global disease burden, accounting for nearly 10% of global deaths.^1^ While some previous studies suggest that light-to-moderate alcohol consumption may reduce the risk of atherosclerosis, these claims are increasingly overshadowed by the mounting evidence of alcohol’s detrimental effects.^2,3,4^

Criticism of alcohol consumption has been steadily accumulating, yet one of the few health-related benefits historically attributed to alcohol is its association with improvements in lipid profiles.^5,6,7^ Small-scale randomized clinical trials and meta-analyses have found that alcohol intake increases high-density lipoprotein cholesterol (HDL-C) and tends to decrease low-density lipoprotein cholesterol (LDL-C).^8,9,10,11,12,13^ However, most interventions in these trials did not reflect lived conditions, as they involved the consumption of more than 3 standard drinks per day, and limited beverage types. Lipid profiles were only measured after a relatively short follow-up period of approximately 1 month after initiation of these interventions. Furthermore, although the health-damaging effects of alcohol have been increasingly emphasized, the effects of alcohol cessation on lipid profiles have not been adequately studied,^14,15^ with observational evidence limited to cross-sectional studies^16,17,18,19,20^ or associations between baseline alcohol intake and subsequent HDL-C change without considering concurrent changes in alcohol consumption.^21^

Given these limitations, it remains unclear whether behavioral, intrapersonal changes in alcohol consumption, particularly alcohol cessation, alter long-term lipid profiles. Therefore, we aimed to elucidate the relationship between alcohol initiation or cessation and subsequent changes in LDL-C and HDL-C, using a large database of longitudinal Japanese annual medical checkups.

## Methods

### Study Setting

The St Luke’s Health Check-up Database (SLHCD) is a large-scale registry that systematically collects data from consecutive participants undergoing annual health checkups at the Center for Preventive Medicine at St Luke’s International Hospital, a tertiary care center in Tokyo, Japan. In Japan, full-time employees of all ages must undergo annual checkups, and employers must encourage all employees to participate in medical checkups.^22^ Details of the SLHCD have been thoroughly documented in previous studies.^23,24,25^ The SLHCD includes demographics, medical and family history, medication use, lifestyle questionnaire data, and medical examination results including blood tests and imaging studies. The hospital’s institutional review board committee approved the study, and all participants provided informed consent through an opt-out process. The study protocol adhered to the Declaration of Helsinki^26^ and the ethical guidelines of the Authoritative Committee on Human Experimentation. This study adheres to the guidelines outlined in the Strengthening the Reporting of Observational Studies in Epidemiology (STROBE) reporting guideline for cohort studies.

### Study Population, Eligibility Criteria, and Cohort Selection

**Figure 1.**
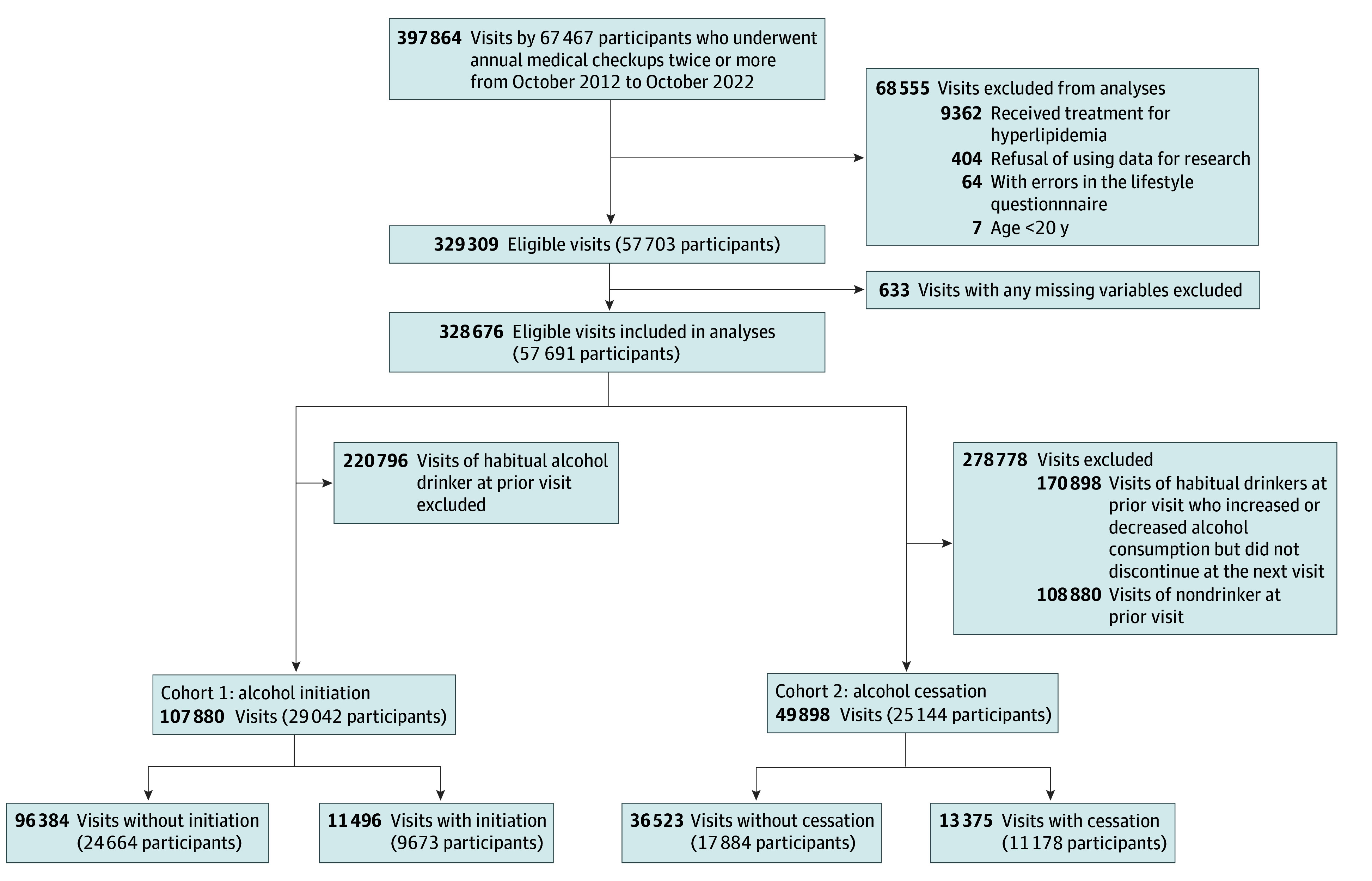
Flowchart of the Study Study population and eligibility criteria are shown.

Participants with data on at least 2 health checkup visits between October 2012 and October 2022 were screened for inclusion in the study. We excluded individuals under 20 years of age (the legal drinking age in Japan), those on lipid-lowering medications, individuals with questionnaire errors, and participants who did not provide research consent (Figure 1). Subsequently, visits with missing values in any variables were excluded, resulting in a dataset comprising 328 676 visits from 57 691 individuals. (Details regarding missing data are provided in eTable 1 in Supplement 1.) We performed additional selection and exclusion of checkup visits, creating the 2 cohorts to evaluate the association of alcohol initiation (cohort 1) and cessation (cohort 2) on changes in LDL-C and HDL-C levels. As all participants had 2 or more visits, we defined each pair of consecutive visits as “prior visit” and “next visit.”

Cohort 1 assessed the association of alcohol initiation on cholesterol levels. We first selected prior visits where participants reported no habitual alcohol consumption. If the participants reported habitual consumption at their next visit, the visits were considered “alcohol initiation visits.” If participants continued to report abstaining from alcohol at the next visit, the visits served as the “no alcohol initiation visits.” Cohort 2 examined the association of alcohol cessation on cholesterol levels by selecting prior visits where participants reported habitual alcohol consumption. If habitual alcohol intake had stopped by the next visit, the visits were labeled as “alcohol cessation visits.” Visits where participants reported the same level of alcohol intake as at the prior visit were considered “alcohol continued visits.”

### Outcome Measures and Exposure Definitions

The primary outcomes were the changes in LDL-C and HDL-C levels reported in milligrams per deciliter (to convert to millimoles per liter, multiply by 0.0259) between 2 consecutive visits. To ensure consistency across all participants, LDL-C and HDL-C levels were routinely measured at each visit in annual health checkups using direct measurement (clinical chemistry analyzers, JCA-BM2250/6070, JEOL Ltd) under standardized laboratory procedures.

The exposure of interest was the change in total daily alcohol intake (standard drinks/d) between 2 consecutive visits. The alcohol intake assessment has been described previously.^23,24,25^ Briefly, a standardized questionnaire was used to collect data on the frequency and average daily intake of alcoholic beverages, including beer, wine, whiskey, sake, shochu (Japanese traditional spirits), and other alcoholic drinks (assumed as distilled spirit-based cocktails based on the National Alcoholic Beverage Survey). We defined 1 standard drink as 10 g of pure ethanol; the corresponding volumes for each beverage are summarized in eTable 2 in Supplement 1. To reduce the influence of outliers, reported alcohol intake was truncated at the 99.9th percentile value, and remaining levels were replaced with that value.

### Covariates

In addition to basic participant characteristics, such as age, sex, and medical history, the health checkup questionnaire collected information regarding lifestyle habits. The frequency of consumption was recorded for carbohydrates (eg, rice, bread, noodles), meat and eggs, seafood, vegetables, fruits, milk and dairy products, soy products, high-fat foods (fried foods, animal fats, and other high-fat foods), and sweet foods. Eating habits, including the frequency of eating until full, eating out, and snacking were documented. Smoking status was categorized as never, former, or current smoker. The frequency of daily physical activity was recorded as very low, low, moderate, or high. Exercise frequency of at least 20 minutes with sweating was categorized as less than 1, 1 to 2, 3 to 5, or more than 5 days per week.

### Statistical Analysis

Continuous variables are shown as mean or median measures, and categorical variables as counts and percentages. Participant-level characteristics at the first visit were summarized according to each cohort. A complete case analysis was performed as the number of missing values was only 633 visits (0.2%). Because each participant contributed multiple visits and thus intrapersonal correlation should be considered, we employed linear generalized estimating equations (GEE) to investigate the association between alcohol initiation or cessation and cholesterol level changes between the 2 visits. Specifically, we specified a Gaussian family with an identity link function to model the continuous changes in cholesterol levels. An exchangeable working correlation structure was used to account for within-participant correlations due to repeated measurements. For cohort 1, model 1 adjusted for the following covariates at the prior visit: age, sex, body mass index (BMI; calculated as weight in kilograms divided by height in meters squared), and LDL-C and HDL-C levels. Model 2 included all covariates from model 1 plus history of hypertension or diabetes, and questionnaire results for dietary habits. The fully adjusted model (model 3) added smoking, daily physical activity, and exercise levels. For cohort 2, we used the same covariates as in cohort 1, and further adjusted for the baseline and frequency of alcohol intake at the prior visit. Because models 1 through 3 included prior cholesterol measurements as covariates, the models for cholesterol levels and those for cholesterol level changes provided the same estimates except for the coefficient of the baseline cholesterol level. Hence, the parameter estimates of alcohol initiation or cessation are interpretable not only as the association with change in cholesterol levels, but also as the association with LDL-C and HDL-C levels.

The primary analysis evaluated the association between continuous changes in total alcohol intake and changes in LDL-C and HDL-C levels, assessing how cholesterol levels changed (mg/dL) with 1-drink alcohol initiation or cessation (drinks/d). Next, we treated the overall alcohol initiation or cessation as a binary exposure variable and evaluated its association with changes in cholesterol levels. The associations between initiation or cessation (drink/d) of individual types of alcoholic beverages and cholesterol changes were then examined. Finally, to minimize the impact of minor fluctuations in alcohol intake, we followed previous meta-analyses and conducted analyses that used categorical alcohol changes as the exposures, with changes in total alcohol intake categorized as fewer than 1.5 drinks/d, 1.5 to 3.0 drinks/d, and 3.0 or more drinks/d.^27^ Stratified subgroup analyses by sex, categorical age, BMI, prior LDL-C, and prior HDL-C were also conducted using model 3. All analyses used R version 4.2.3 (R Foundation for Statistical Computing), and point estimates with 95% confidence intervals (CIs) were reported for all analyses. Because we have conducted multiple analyses to show the robustness of the results, we provided the 99% CIs to account for the simultaneous estimation of the associations for 6 exposure variables (ie, alcohol types), where 99% is roughly calculated from *1 − 0.05 / 6 = 99.2%*. The threshold for significance is 2-sided *P* < .05.

## Results

### Participant Characteristics

Analysis included 328 676 visits from 57 691 individuals (30 576 female [53.0%]). Cohort 1 comprised 107 880 visits (29 042 participants; mean [SD] age, 47.0 [13.6] years; 19 269 female [66.3%]) (Table 1). Prior mean (SD) LDL-C and HDL-C levels were 116.4 (28.6) mg/dL and 63.6 (15.4) mg/dL, respectively. At initiation visits, the median (IQR) alcohol intake was 0.40 (0.20-0.80) drinks/d.

**Table 1.** Participant-Level Characteristics at the First Visit

Characteristic	Cohort 1, Participants, No. (%)		Cohort 2, Participants, No. (%)	
	No alcohol initiation (n = 24 664)^a^	Alcohol initiation (n = 9673)^a^	Alcohol continued (n = 17 884)^a^	Alcohol cessation (n = 11 178)^a^
Age, mean (SD), y	47.4 (13.7)	46.0 (13.2)	50.3 (11.4)	47.6 (13.2)
Sex				
Female	16 750 (67.9)	5853 (60.5)	8143 (45.5)	6391 (57.2)
Male	7914 (32.1)	3820 (39.5)	9741 (54.5)	4787 (42.8)
BMI, mean (SD)	21.8 (3.5)	22.0 (3.4)	22.2 (3.2)	22.2 (3.5)
Interval between visits, median (IQR), y	1.01 (0.98-1.15)	1.01 (0.97-1.11)	1.00 (0.97-1.07)	1.02 (0.98-1.21)
Prior LDL-Cholesterol, mean (SD), mg/dL	116.9 (28.4)	115.6 (28.7)	114.5 (28.3)	116.3 (28.6)
Prior HDL-Cholesterol, mean (SD), mg/dL	63.6 (15.4)	63.9 (15.7)	66.4 (16.6)	64.2 (16.1)
Hypertension	1576 (6.4)	642 (6.6)	1860 (10.4)	837 (7.5)
Diabetes	624 (2.5)	225 (2.3)	426 (2.4)	288 (2.6)
Alcohol data at first visit				
Total alcohol intake, standard drink, median (IQR), No./d	NA	NA	1.4 (0.6-2.8)	0.4 (0.2-0.8)
Frequency of alcohol intake, median (IQR), d/wk	NA	NA	4 (2-7)	1 (1-2)
Beer drinker	NA	NA	12 160 (68.0)	6988 (62.5)
Daily intake, median (IQR), standard drink/d	NA	NA	1.1 (0.4-1.4)	0.4 (0.2-0.7)
Wine drinker	NA	NA	4828 (27.0)	3040 (27.2)
Daily intake, median (IQR), standard drink/d	NA	NA	1.0 (0.5-2.0)	0.3 (0.2-0.7)
Whiskey drinker	NA	NA	688 (3.8)	452 (4.0)
Daily intake, median (IQR), standard drink/d	NA	NA	1.6 (0.8-2.5)	0.4 (0.3-1.0)
Shochu drinker	NA	NA	2235 (12.5)	1007 (9.0)
Daily intake, median (IQR), standard drink/d	NA	NA	3.4 (1.7-5.1)	0.9 (0.3-1.7)
Sake drinker	NA	NA	1277 (7.1)	568 (5.1)
Daily intake, median (IQR), standard drink/d	NA	NA	2.2 (1.2-3.7)	0.6 (0.3-1.2)
Other drinker	NA	NA	313 (1.8)	778 (7.0)
Daily intake, median (IQR), standard drink/d	NA	NA	0.6 (0.3-1.7)	0.6 (0.3-0.8)

Abbreviations: BMI, body mass index (calculated as weight in kilograms divided by height in meters squared); HDL-C, high-density lipoprotein cholesterol; LDL-C, low-density lipoprotein cholesterol; NA, not applicable.

SI conversion factor: To convert cholesterol to millimoles per liter, multiply by 0.0259.

^a^ Because the analysis was conducted at the visit level, the same patient could be included in both groups.

Cohort 2 comprised 49 898 visits (25 144 participants; mean [SD] age, 48.9 [12.1] years; 12 334 female [49.1%]). Prior mean (SD) LDL-C and HDL-C levels were 114.7 (28.4) mg/dL and 65.5 (16.4) mg/dL, respectively. The participants ceased alcohol intake from the median (IQR) intake of 0.40 (0.20-0.80) drinks/d. Lifestyle and dietary data are shown in eTable 3 in Supplement 1.

### Association Between Alcohol Initiation and Change in LDL-C and HDL-C (Cohort 1)

**Figure 2.**
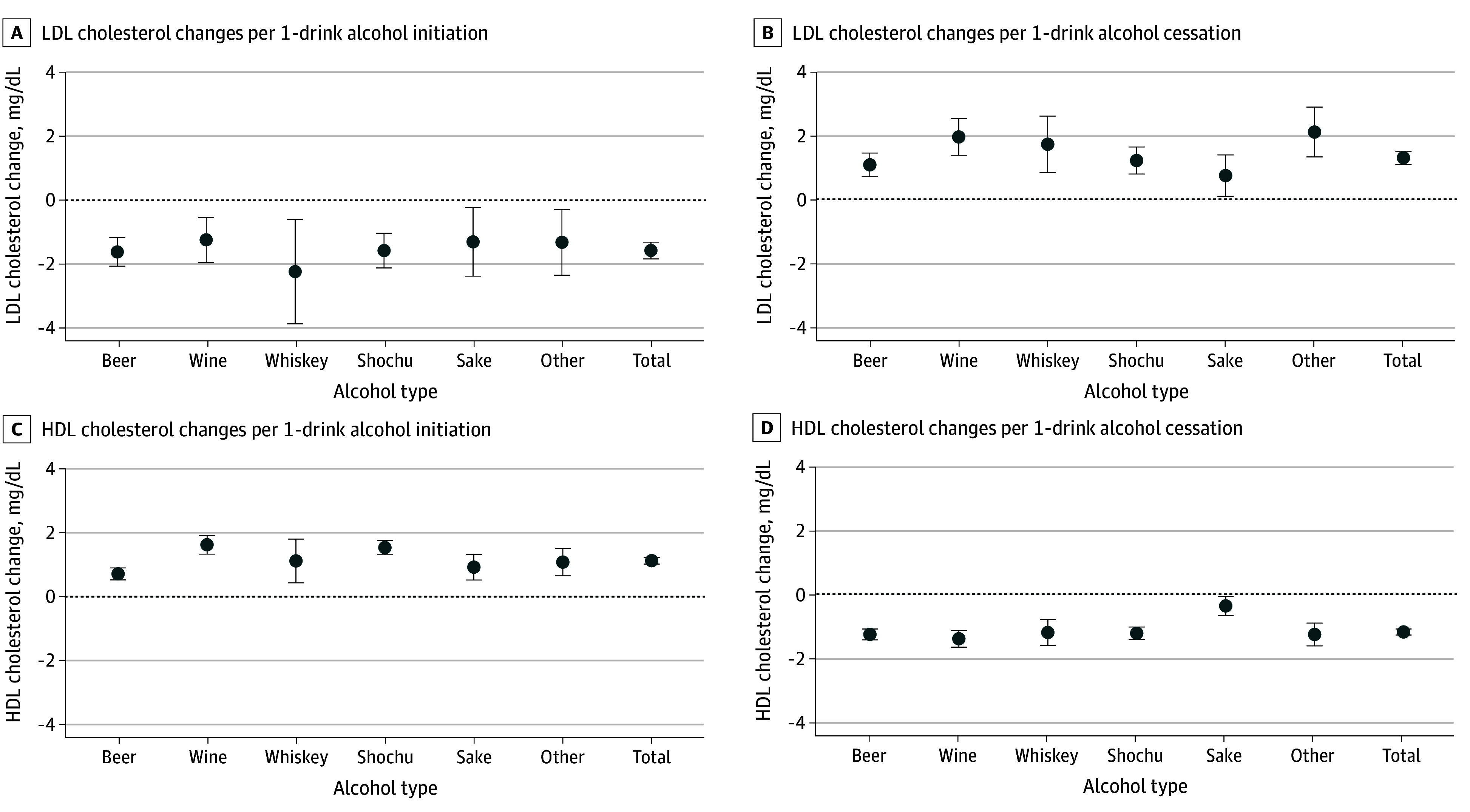
Association of Beverage-Specific Alcohol Consumption and Changes in Lipid Concentration Association of specific alcoholic beverages with changes in lipid concentrations. Results are shown from the fully adjusted model (Model 3). LDL indicates low-density lipoprotein; HDL, high-density lipoprotein. To convert cholesterol to millimoles per liter, multiply by 0.0259.

Initiating an additional 1 alcoholic drink/d was associated with decreased LDL-C levels and increased HDL-C levels, with similar results across the 3 models (Table 2; eTable 4 in Supplement 1). Initiation of all types of alcoholic beverages was similarly associated with decreased LDL-C and increased HDL-C levels (Figure 2).

**Table 2.** Multivariable-Adjusted Models Evaluating the Association Between Initiating and Ceasing Alcohol Consumption and Changes in Lipid Concentration

Exposure	β (95% CI)		
	Model 1^a^	Model 2^b^	Model 3^c^
**Alcohol initiation (cohort 1)**			
LDL-C change			
1-drink increase of alcohol	−1.58 (−1.83 to −1.32)	−1.60 (−1.86 to −1.35)	−1.59 (−1.84 to −1.33)
Overall initiation	−1.26 (−1.58 to −0.94)	−1.29 (−1.60 to −0.97)	−1.27 (−1.59 to −0.95)
HDL-C change			
1-drink increase of alcohol	1.14 (1.03 to 1.25)	1.15 (1.04 to 1.25)	1.15 (1.04 to 1.26)
Overall initiation	0.84 (0.71 to 0.97)	0.85 (0.72 to 0.99)	0.85 (0.72 to 0.99)
**Alcohol cessation (cohort 2)** ^d^			
LDL-C change			
1-drink decrease of alcohol	1.39 (1.17 to 1.60)	1.41 (1.20 to 1.63)	1.41 (1.20 to 1.63)
Overall cessation	1.41 (1.05 to 1.77)	1.48 (1.12 to 1.83)	1.48 (1.12 to 1.84)
HDL-C change			
1-drink decrease of alcohol	−1.26 (−1.36 to −1.16)	−1.25 (−1.35 to −1.16)	−1.25 (−1.36 to −1.16)
Overall cessation	−1.62 (−1.78 to −1.46)	−1.59 (−1.75 to −1.42)	−1.59 (−1.75 to −1.42)

Abbreviations: BMI, body mass index (calculated as weight in kilograms divided by height in meters squared); HDL-C, high-density lipoprotein cholesterol; LDL-C, low-density lipoprotein cholesterol.

^a^ Adjusted for age, sex, BMI, and prior LDL-C and HDL-C cholesterol.

^b^ Adjusted for covariates in model 1 plus history of hypertension, history of diabetes, dietary habits (frequency of carbohydrates, meat and eggs, seafood, vegetables, fruits, milk and dairy products, soy, fat-rich diet, and sweets; frequency of eating until full, eating out, and snacks between meals).

^c^ Adjusted for covariates in model 2 plus smoking, daily physical activity, and exercise level.

^d^ In cohort 2, analyses additionally adjusted for the amount of alcohol intake and frequency of alcohol intake.

**Figure 3.**
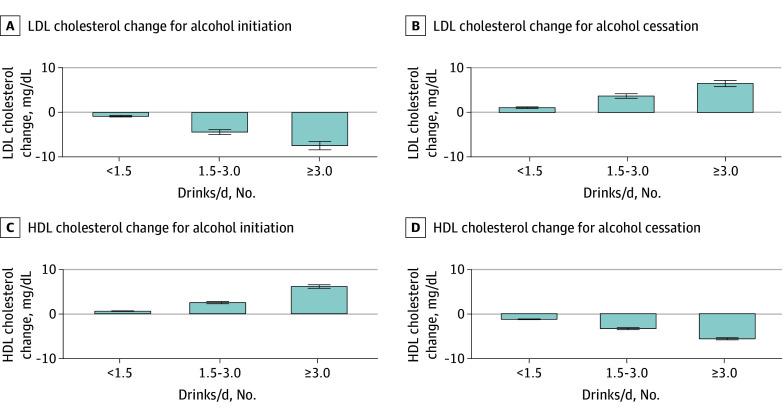
Adjusted Association Between Initiating and Ceasing Alcohol Consumption (Categorical) and Changes in Lipid Concentration Adjusted association of categorized alcohol initiation and cessation with changes in lipid concentrations. Alcohol intake is categorized as fewer than 1.5 drinks/d, 1.5 to 3.0 drinks/d, and 3.0 or more drinks/d. Results are presented from the fully adjusted model (model 3). LDL indicates low-density lipoprotein; HDL, high-density lipoprotein. To convert cholesterol to millimoles per liter, multiply by 0.0259.

When alcohol intake changes were categorized, the adjusted mean changes in LDL-C levels with alcohol initiation were −0.85 mg/dL (95% CI, −1.18 to −0.52 mg/dL) in participants initiating fewer than 1.5 alcohol drinks/d, −4.40 mg/dL (95% CI, −5.52 to −3.29 mg/dL) with 1.5 to 3.0 drinks/d, and −7.44 mg/dL (95% CI, −9.29 to −5.60 mg/dL) with 3.0 or more drinks/d compared with noninitiators. Similarly, initiation of alcohol consumption was associated with increased HDL-C levels (fewer than 1.5 alcohol drinks/d, 0.58 mg/dL [95% CI, 0.44 to 0.72 mg/dL]; 1.5 to 3.0 drinks/d, 2.49 mg/dL [95% CI, 2.02 to 2.96 mg/dL]; and 3.0 or more drinks/d, 6.12 mg/dL [95% CI, 5.34 to 6.90 mg/dL]) (Figure 3).

In stratified subgroup analyses, female participants, those with lower BMI, and those with higher prior HDL-C levels had larger decreases in LDL-C levels, whereas older participants, male participants, and those with higher prior LDL-C levels had larger increases in HDL-C levels (eFigures 1 and 2 in Supplement 1).

### Association Between Alcohol Cessation and Change in LDL-C and HDL-C (Cohort 2)

Cessation of 1 alcohol drink was associated with increased LDL-C and decreased HDL-C levels (Table 2; eTable 4 in Supplement 1). Cessation of all types of alcoholic beverages was associated with increased LDL-C and decreased HDL-C levels (Figure 2).

When alcohol intake changes were categorized, the adjusted mean changes in LDL-C levels with alcohol cessation were 1.10 mg/dL (95% CI, 0.76 to 1.45 mg/dL) in participants discontinuing habits of fewer than 1.5 alcohol drinks/d, −3.71 mg/dL (95% CI, −2.71 to −4.71 mg/dL) with 1.5 to 3.0 drinks/d, and 6.53 mg/dL (95% CI, 5.14 to 7.91 mg/dL) with 3.0 or more drinks/d, compared with those who continued alcohol consumption. Similarly, cessation of alcohol consumption was associated with decreased HDL-C (fewer than 1.5 drinks/d, −1.25 mg/dL [95% CI, −1.41 to −1.09 mg/dL]), 1.5 to 3.0 drinks/d (−3.35 [95% CI, −4.41 to −2.29 mg/dL]), and 3.0 or more drinks/d (−5.65 mg/dL [95% CI, −6.28 to −5.01 mg/dL]) (Figure 3).

In subgroup analyses, alcohol cessation was associated with increased LDL-C levels among younger participants, female participants, and those with lower BMI, lower prior LDL-C, and higher prior HDL-C, whereas decreased HDL-C levels were associated with older age, lower BMI, lower prior LDL-C, and higher prior HDL-C (eFigures 3 and 4 in Supplement 1).

## Discussion

The current study found that alcohol initiation was associated with decreased LDL-C and increased HDL-C levels, whereas alcohol cessation showed an opposite association in a clinical setting. These changes were more pronounced with higher amounts of alcohol consumption in a dose-dependent manner. Although the mechanisms by which alcohol affects cholesterol levels remain unclear,^27^ similar associations with lipid profiles across various alcoholic beverages suggest that alcohol is the primary causative substance.

Unlike previous observational studies, our study uniquely focused on the association between changes in exposure and subsequent outcomes, such as alcohol initiation and cholesterol changes. This design may have helped us replicate the results from previous interventional studies within our observational dataset. Our study found that initiating an ethanol intake of 15 to 30 g/d was associated with increased HDL-C levels by 2.49 mg/dL, while a 30 g/d increase or more was associated with increased HDL-C levels by 6.12 mg/dL. These results are consistent with previous meta-analyses reporting that daily ethanol intake of 30 g was associated with increased HDL-C by 3.66 mg/dL^27^ and 3.99 mg/dL.^6^ Consistent with previous research, stratified analyses showed alcohol initiation affects lipid profiles, with greater changes observed in women and individuals with lower BMI who discontinue consumption.^28^ Most clinical trials observed changes in lipid profiles over a short period of approximately 1 month, and were limited to specific types of alcoholic beverages. Our study extended the follow-up period up to 1 year and assessed a wider range of alcoholic beverages, substantially expanding the existing evidence. Integrating these results, our findings support the hypothesis that alcohol consumption may improve lipid profile and prevent specific diseases, such as atherosclerosis, even from a longer-term perspective.

More importantly, this study revealed that alcohol intake cessation was associated with increased LDL-C and decreased HDL-C levels. This has not been fully examined despite increasing concerns about alcohol intake and overall risks. In a small number of alcohol-dependent patients, alcohol cessation is associated with reduced insulin-like growth factor-binding protein-1 (IGFBP-1) and increased lipoprotein(a), with the reduction in IGFBP-1 being known to correlate with decreased HDL-C levels.^29^ A 4-week alcohol abstinence study demonstrated significantly decreased HDL-C and elevated LDL-C–to–HDL-C ratios,^30^ consistent with our study. Current evidence emphasizes the harmfulness of alcohol consumption and the importance of actively reducing alcohol consumption, especially among heavier drinkers.^31,32^ In this study, alcohol cessation was associated with worsening lipid profiles even among non-heavy drinkers. The HDL-C changes induced by alcohol in this study exceeded what is typically achievable through therapeutic interventions, including fibrate therapy.^33^ While the absolute LDL-C change appears modest, a 5 mg/dL increase in LDL-C is associated with a 2% to 3% elevated risk of major cardiovascular events, suggesting considerable public health impacts at the population level.^34^ It is worth emphasizing that alcohol consumption is associated with increased triglycerides, high blood pressure, arrhythmia, and cardiomyopathy.^35^ This highlights the delicate balance between maintaining healthy cholesterol profiles and other alcohol-associated risks. Considering current international efforts to regulate moderate alcohol intake to mitigate overall health risks, this study emphasizes the importance of carefully monitoring cholesterol levels after alcohol cessation to optimize lipid profile management at both the individual and population levels.

Other lifestyle factors than alcohol, such as obesity, exercise, smoking, and dietary habits, may also affect cholesterol levels.^36^ Individuals who consume alcohol tend to be obese and have distinctive dietary patterns characterized by lower intake of carbohydrates and sweets and higher consumption of fish, a pattern that was observed in our study.^37^ While low-carbohydrate diets reduce body weight compared with low-fat diets, they have potential adverse effects on cholesterol, such as increasing LDL-C levels.^38,39^ The impact of lifestyle changes regarding alcohol consumption on lipid profiles needs to be considered within this complex interplay of factors. This study comprehensively adjusted for these lifestyle factors, including detailed dietary components assessed at prior visits. Additionally, its longitudinal design, which examined intrapersonal changes over time, accounts for variations in lifestyle among study participants. However, our analyses did not consider other lifestyle changes that concurrently occurred with alcohol intake changes, because these factors could serve as either confounders or mediators. For instance, if alcohol cessation is accompanied by dietary or lifestyle changes that improve the lipid profile, this study may underestimate the effect of alcohol cessation. Comprehensive lifestyle management would be crucial for cardiovascular risk reduction in clinical practice.

### Limitations

This study had several limitations. First, our study relied on self-reported alcohol intake, which may be subject to recall and social desirability biases. Participants may have underreported their alcohol consumption or misclassified their drinking patterns. However, we used data from a standardized questionnaire to calculate alcohol intake based on specific beverage types, which may improve accuracy. Additionally, the longitudinal nature of our study may have mitigated some of these biases, as we focused on changes in alcohol consumption. Second, although we adjusted for comprehensive covariates, including demographic factors, medical history, and lifestyle habits, residual confounding factors could not be completely ruled out. For instance, we may not have captured all aspects of diet quality or stress levels that could influence lipid profiles; however, the consistency of the results across multiple adjustment models reinforces the robustness of our findings. Third, while our study population was predominantly Japanese, which limits generalizability owing to potential genetic and cultural differences in alcohol metabolism, the consistency of our findings with those of international studies suggests shared biological mechanisms across populations. Fourth, although we observed lipid profile changes, we did not assess hard cardiovascular outcomes such as myocardial infarction or stroke. However, cholesterol is an established risk factor and management target for cardiovascular disease (CVD). Fifth, multiple analyses in this study may increase the risk of type I error. Although the results should be carefully interpreted given the potential false positives, our main findings remained robust under a more conservative 99% CI.

## Conclusions

In this cohort study of Japanese health checkup participants, both the initiation and cessation of alcohol consumption were associated with changes in LDL-C and HDL-C levels in a dose-dependent manner. While light alcohol consumption may have potentially favorable effects on lipid profiles, decisions regarding alcohol intake should be individualized, considering the overall health risks. Public health recommendations should continue to emphasize moderation in alcohol consumption, but cholesterol levels should be carefully monitored after alcohol cessation to mitigate potential CVD risks.

## Notes

Supplement 1. **eTable 1.** Missing Information on Visit-Level Participants’ Characteristics **eTable 2.** Standard Drink Volume for Each Alcoholic Beverage **eTable 3.** Participant-Level Lifestyle Questionnaire Results **eTable 4.** Multivariable Adjusted Models Evaluating the Association Between Initiating and Ceasing Alcohol Consumption and Changes in Lipid Concentration With 99% Confidence Intervals **eFigure 1.** Subgroup Analysis of Adjusted Association Between Initiating Alcohol Consumption (Categorical) and Changes in LDL Cholesterol **eFigure 2.** Subgroup Analysis of Adjusted Association Between Initiating Alcohol Consumption (Categorical) and Changes in HDL Cholesterol **eFigure 3.** Subgroup Analysis of Adjusted Association Between Ceasing Alcohol Consumption (Categorical) and Changes in LDL Cholesterol **eFigure 4.** Subgroup Analysis of Adjusted Association Between Ceasing Alcohol Consumption (Categorical) and Changes in HDL Cholesterol

Supplement 2.
Data Sharing Statement

## References

[1] GBD 2016 Alcohol Collaborators. Alcohol use and burden for 195 countries and territories, 1990-2016: a systematic analysis for the Global Burden of Disease Study 2016. Lancet. 2018;392(10152):1015-1035. doi:10.1016/S0140-6736(18)31310-2 30146330 PMC6148333

[2] Bell S, Daskalopoulou M, Rapsomaniki E, . Association between clinically recorded alcohol consumption and initial presentation of 12 cardiovascular diseases: population based cohort study using linked health records. BMJ. 2017;356:j909. doi:10.1136/bmj.j909 28331015 PMC5594422

[3] O’Keefe JH, Bybee KA, Lavie CJ. Alcohol and cardiovascular health: the razor-sharp double-edged sword. J Am Coll Cardiol. 2007;50(11):1009-1014. doi:10.1016/j.jacc.2007.04.089 17825708

[4] Visontay R, Sunderland M, Slade T, Wilson J, Mewton L. Are there non-linear relationships between alcohol consumption and long-term health? Protocol for a systematic review of observational studies employing approaches to improve causal inference. BMJ Open. 2021;11(3):e043985. doi:10.1136/bmjopen-2020-043985 33757947 PMC7993196

[5] Jackson R, Scragg R, Beaglehole R. Alcohol consumption and risk of coronary heart disease. BMJ. 1991;303(6796):211-216. doi:10.1136/bmj.303.6796.211 1884056 PMC1670516

[6] Rimm EB, Williams P, Fosher K, Criqui M, Stampfer MJ. Moderate alcohol intake and lower risk of coronary heart disease: meta-analysis of effects on lipids and haemostatic factors. BMJ. 1999;319(7224):1523-1528. doi:10.1136/bmj.319.7224.1523 10591709 PMC28294

[7] Waśkiewicz A, Sygnowska E. Alcohol intake and cardiovascular risk factor profile in men participating in the WOBASZ study. Kardiol Pol. 2013;71(4):359-365. doi:10.5603/KP.2013.0063 23788341

[8] Chiva-Blanch G, Urpi-Sarda M, Ros E, . Effects of red wine polyphenols and alcohol on glucose metabolism and the lipid profile: a randomized clinical trial. Clin Nutr. 2013;32(2):200-206. doi:10.1016/j.clnu.2012.08.022 22999066

[9] Padro T, Muñoz-García N, Vilahur G, . Moderate beer intake and cardiovascular health in overweight individuals. Nutrients. 2018;10(9):1237. doi:10.3390/nu10091237 30189619 PMC6164820

[10] Park H, Kim K. Relationship between alcohol consumption and serum lipid levels in elderly Korean men. Arch Gerontol Geriatr. 2012;55(2):226-230. doi:10.1016/j.archger.2011.08.014 21925744

[11] Mori TA, Burke V, Beilin LJ, Puddey IB. Randomized controlled intervention of the effects of alcohol on blood pressure in premenopausal women. Hypertension. 2015;66(3):517-523. doi:10.1161/HYPERTENSIONAHA.115.05773 26123682

[12] Chiva-Blanch G, Magraner E, Condines X, . Effects of alcohol and polyphenols from beer on atherosclerotic biomarkers in high cardiovascular risk men: a randomized feeding trial. Nutr Metab Cardiovasc Dis. 2015;25(1):36-45. doi:10.1016/j.numecd.2014.07.008 25183453

[13] Kechagias S, Zanjani S, Gjellan S, . Effects of moderate red wine consumption on liver fat and blood lipids: a prospective randomized study. Ann Med. 2011;43(7):545-554. doi:10.3109/07853890.2011.588246 21599573

[14] Haskell WL, Camargo C Jr, Williams PT, . The effect of cessation and resumption of moderate alcohol intake on serum high-density-lipoprotein subfractions—A controlled study. N Engl J Med. 1984;310(13):805-810. doi:10.1056/NEJM198403293101301 6366553

[15] Romeo J, González-Gross M, Wärnberg J, Díaz LE, Marcos A. Effects of moderate beer consumption on blood lipid profile in healthy Spanish adults. Nutr Metab Cardiovasc Dis. 2008;18(5):365-372. doi:10.1016/j.numecd.2007.03.007 17976963

[16] Timon R, Olcina G, Maynar JI, Maynar M. Effects of regular and abusive intake of alcohol at weekends on physiological parameters in Spanish young. Public Health. 2012;126(10):873-880. doi:10.1016/j.puhe.2012.06.004 23025982

[17] Hao G, Wang Z, Zhang L, . Relationship between alcohol consumption and serum lipid profiles among middle-aged population in China: a multiple-center cardiovascular epidemiological study. Angiology. 2015;66(8):753-758. doi:10.1177/0003319714549557 25192699

[18] Kwon YJ, Kim SE, Park BJ, Bae JW, Kang HT. High-risk drinking is associated with dyslipidemia in a different way, based on the 2010-2012 KNHANES. Clin Chim Acta. 2016;456:170-175. doi:10.1016/j.cca.2016.03.009 27000703

[19] Shen Z, Munker S, Wang C, . Association between alcohol intake, overweight, and serum lipid levels and the risk analysis associated with the development of dyslipidemia. J Clin Lipidol. 2014;8(3):273-278. doi:10.1016/j.jacl.2014.02.003 24793348

[20] Perissinotto E, Buja A, Maggi S, ; ILSA Working Group. Alcohol consumption and cardiovascular risk factors in older lifelong wine drinkers: the Italian Longitudinal Study on Aging. Nutr Metab Cardiovasc Dis. 2010;20(9):647-655. doi:10.1016/j.numecd.2009.05.014 19695851

[21] Huang S, Li J, Shearer GC, . Longitudinal study of alcohol consumption and HDL concentrations: a community-based study. Am J Clin Nutr. 2017;105(4):905-912. doi:10.3945/ajcn.116.144832 28251934 PMC5366050

[22] OECD. Health check-ups in japan. In: OECD Reviews of Public Health. OECD; 2019:127-170.

[23] Fukui S, Okada M, Rahman M, . Differences in the association between alcoholic beverage type and serum urate levels using standardized ethanol content. JAMA Netw Open. 2023;6(3):e233398. doi:10.1001/jamanetworkopen.2023.3398 36930152 PMC10024203

[24] Fukui S, Okada M, Shinozaki T, . Changes in alcohol intake and serum urate changes: longitudinal analyses of annual medical examination database. Ann Rheum Dis. 2024;83(8):1072-1081. doi:10.1136/ard-2023-225389 38418204 PMC11250628

[25] Fukui S, Okada M, Shinozaki T, . Weight reduction and target serum urate level: a longitudinal study of annual medical examination. Arthritis Rheumatol. Published online October 14, 2024. doi:10.1002/art.43027 39400956 PMC11867883

[26] World Medical Association. World Medical Association Declaration of Helsinki: ethical principles for medical research involving human subjects. JAMA. 2013;310(20):2191-2194. doi:10.1001/jama.2013.281053 24141714

[27] Brien SE, Ronksley PE, Turner BJ, Mukamal KJ, Ghali WA. Effect of alcohol consumption on biological markers associated with risk of coronary heart disease: systematic review and meta-analysis of interventional studies. BMJ. 2011;342:d636. doi:10.1136/bmj.d636 21343206 PMC3043110

[28] Weidner G, Connor SL, Chesney MA, . Sex differences in high density lipoprotein cholesterol among low-level alcohol consumers. Circulation. 1991;83(1):176-180. doi:10.1161/01.CIR.83.1.176 1984880

[29] Paassilta M, Kervinen K, Linnaluoto M, Kesäniemi YA. Alcohol withdrawal-induced change in lipoprotein(a): association with the growth hormone/insulin-like growth factor-I (IGF-I)/IGF-binding protein-1 (IGFBP-1) axis. Arterioscler Thromb Vasc Biol. 1998;18(4):650-654. doi:10.1161/01.ATV.18.4.650 9555872

[30] Huang CM, Elin RJ, Ruddel M, Schmitz J, Linnoila M. The effect of alcohol withdrawal on serum concentrations of Lp(a), apolipoproteins A-1 and B, and lipids. Alcohol Clin Exp Res. 1992;16(5):895-898. doi:10.1111/j.1530-0277.1992.tb01889.x 1332524

[31] Biddinger KJ, Emdin CA, Haas ME, . Association of habitual alcohol intake with risk of cardiovascular disease. JAMA Netw Open. 2022;5(3):e223849. doi:10.1001/jamanetworkopen.2022.3849 35333364 PMC8956974

[32] Wood AM, Kaptoge S, Butterworth AS, ; Emerging Risk Factors Collaboration/EPIC-CVD/UK Biobank Alcohol Study Group. Risk thresholds for alcohol consumption: combined analysis of individual-participant data for 599 912 current drinkers in 83 prospective studies. Lancet. 2018;391(10129):1513-1523. doi:10.1016/S0140-6736(18)30134-X 29676281 PMC5899998

[33] Briel M, Ferreira-Gonzalez I, You JJ, . Association between change in high density lipoprotein cholesterol and cardiovascular disease morbidity and mortality: systematic review and meta-regression analysis. BMJ. 2009;338:b92. doi:10.1136/bmj.b92 19221140 PMC2645847

[34] Silverman MG, Ference BA, Im K, . Association between lowering LDL-C and cardiovascular risk reduction among different therapeutic interventions: a systematic review and meta-analysis. JAMA. 2016;316(12):1289-1297. doi:10.1001/jama.2016.13985 27673306

[35] Di Federico S, Filippini T, Whelton PK, . Alcohol intake and blood pressure levels: a dose-response meta-analysis of nonexperimental cohort studies. Hypertension. 2023;80(10):1961-1969. doi:10.1161/HYPERTENSIONAHA.123.21224 37522179 PMC10510850

[36] Barnard RJ. Effects of life-style modification on serum lipids. Arch Intern Med. 1991;151(7):1389-1394. doi:10.1001/archinte.1991.00400070141019 2064490

[37] Tanisawa K, Ito T, Kawakami R, . Association between alcohol dietary pattern and prevalence of dyslipidaemia: WASEDA’S Health Study. Br J Nutr. 2022;127(11):1712-1722. doi:10.1017/S0007114521002671 34256880 PMC9201834

[38] Mansoor N, Vinknes KJ, Veierød MB, Retterstøl K. Effects of low-carbohydrate diets v. low-fat diets on body weight and cardiovascular risk factors: a meta-analysis of randomised controlled trials. Br J Nutr. 2016;115(3):466-479. doi:10.1017/S0007114515004699 26768850

[39] Retterstøl K, Svendsen M, Narverud I, Holven KB. Effect of low carbohydrate high fat diet on LDL cholesterol and gene expression in normal-weight, young adults: a randomized controlled study. Atherosclerosis. 2018;279:52-61. doi:10.1016/j.atherosclerosis.2018.10.013 30408717

