# Error in Article Information

**DOI:** 10.1001/jamanetworkopen.2025.15488

**Published:** 2025-05-09

In the Original Investigation titled “Lipid Profiles After Changes in Alcohol Consumption Among Adults Undergoing Annual Checkups,”^1^ published March 12, 2025, there was an error in degrees listed for contributors in the Additional Contributions section. This article has been corrected.^1^

## References

[1] Suzuki T, Fukui S, Shinozaki T, . Lipid profiles after changes in alcohol consumption among adults undergoing annual checkups. JAMA Netw Open. 2025;8(3):e250583. doi:10.1001/jamanetworkopen.2025.0583 40072433 PMC11904732

