# Supplementary material for: Lipid Profiles After Changes in Alcohol Consumption Among Adults Undergoing Annual Checkups
Source: JAMA Netw Open. 2025 Mar 12;8(3):e250583. doi: 10.1001/jamanetworkopen.2025.0583 (PMC11904732; doi:10.1001/jamanetworkopen.2025.0583)
Supplement: Supplement 1. — eTable 1. Missing Information on Visit-Level Participants’ Characteristics eTable 2. Standard Drink Volume for Each Alcoholic Beverage eTable 3. Participant-Level Lifestyle Questionnaire Results eTable 4. Multivariable Adjusted Models Evaluating the Association Between Initiating and Ceasing Alcohol Consumption and Changes in Lipid Concentration With 99% Confidence Intervals eFigure 1. Subgroup Analysis of Adjusted Association Between Initiating Alcohol Consumption (Categorical) and Changes in LDL Cholesterol eFigure 2. Subgroup Analysis of Adjusted Association Between Initiating Alcohol Consumption (Categorical) and Changes in HDL Cholesterol eFigure 3. Subgroup Analysis of Adjusted Association Between Ceasing Alcohol Consumption (Categorical) and Changes in LDL Cholesterol eFigure 4. Subgroup Analysis of Adjusted Association Between Ceasing Alcohol Consumption (Categorical) and Changes in HDL Cholesterol [file jamanetwopen-e250583-s001.pdf]

Supplemental Online Content

Suzuki T, Fukui S, Shinozaki T, et al. Lipid profiles after changes in alcohol consumption among adults undergoing annual checkups. *JAMA Netw Open.* 2025;8(3):e250583. doi:10.1001/jamanetworkopen.2025.0583

eTable 1. Missing Information on Visit-Level Participants' Characteristics

eTable 2. Standard Drink Volume for Each Alcoholic Beverage

eTable 3. Participant-Level Lifestyle Questionnaire Results

eTable 4. Multivariable Adjusted Models Evaluating the Association Between Initiating and Ceasing Alcohol Consumption and Changes in Lipid Concentration With 99% Confidence Intervals

eFigure. Subgroup Analysis of Adjusted Association Between Alcohol Consumption (Categorical) and Changes in Cholesterol

This supplemental material has been provided by the authors to give readers additional information about their work.

© 2025 Suzuki T et al. *JAMA Network Open*.

eTable 1. Missing information on visit-level participants' characteristics

Variables	Number of missing values, No. (%) (Among 329,309 visits)
Age	0 (0)
Female gender	0 (0)
Body Mass Index	19 (0.01)
Interval between visits	0 (0)
Prior LDL-cholesterol	29 (0.01)
Prior HDL-cholesterol	29 (0.01)
Hypertension	0 (0)
Diabetes	0 (0)
Alcohol information	0 (0)
Smoke	0 (0)
Daily Physical Activity	0 (0)
Exercise	0 (0)
Diet	
Carbohydrate	603 (0.18)
Meat and Eggs	609 (0.18)
Seafood	605 (0.18)
Soy	607 (0.18)
Milk and daily product	602 (0.18)
Vegetables	609 (0.18)
Fruits	606 (0.18)
Sweets	597 (0.18)
Fatty Diet	603 (0.18)
Eating until becoming full	588 (0.18)
Eating out	595 (0.18)
Snacks between meals	592 (0.18)

© 2025 Suzuki T et al. *JAMA Network Open*.

eTable 2. Standard drink volume for each alcoholic beverage

Beverage	Volume [mL]	Approximate Equivalent
Beer	250	-
Wine	104	0.9 glasses
Sake	84	0.47 of a Japanese traditional sake bottle
Shochu	50	0.5 glass
Whiskey	31	1 shot

© 2025 Suzuki T et al. *JAMA Network Open*.

eTable 3. Participant-level lifestyle questionnaire results

Characteristic		Cohort 1		Cohort 2	
Participants, No. (%)		No alcohol-initiation cohort 24,664 participants	Alcohol-initiation cohort 9,673 participants	Alcohol-continued cohort 17,884 participants	Alcohol cessation cohort 11,178 participants
Questionnaire Lifestyle					
Smoke	Never	19063 (77.3)	7089 (73.3)	10600 (59.3)	7879 (70.5)
	Previous	3897 (15.8)	1874 (19.4)	5208 (29.1)	2374 (21.2)
	Current	1704 (6.9)	710 (7.3)	2076 (11.6)	925 (8.3)
Daily Physical Activity	Very low	3121 (12.7)	1181 (12.2)	1756 (9.8)	1336 (12.0)
	Low	8043 (32.6)	3193 (33.0)	5688 (31.8)	3861 (34.5)
	Moderate	11601 (47.0)	4524 (46.8)	8790 (49.2)	5129 (45.9)
	High	1899 (7.7)	775 (8.0)	1650 (9.2)	852 (7.6)
Exercise	Almost none	9098 (36.9)	3424 (35.4)	5110 (28.6)	3710 (33.2)
	1-2 days / week	8459 (34.3)	3505 (36.2)	7220 (40.4)	4299 (38.5)
	3-5 days / week	4568 (18.5)	1807 (18.7)	3551 (19.9)	2081 (18.6)
	Almost everyday	2539 (10.3)	937 (9.7)	2003 (11.2)	1088 (9.7)
Diet					
Carbohydrate	Less than 1 serving/day	318 (1.3)	170 (1.8)	299 (1.7)	201 (1.8)
	1 serving/day	2327 (9.4)	1163 (12.0)	2910 (16.3)	1511 (13.5)
	2 servings/day	6073 (24.6)	2666 (27.6)	5715 (32.0)	3171 (28.4)
	≥ 3 servings/day	15946 (64.7)	5674 (58.7)	8960 (50.1)	6295 (56.3)
Meat and Eggs	Less frequently	391 (1.6)	95 (1.0)	119 (0.7)	95 (0.8)
	1-2 days/week	4713 (19.1)	1688 (17.5)	3342 (18.7)	1977 (17.7)
	3-4 days/week	13722 (55.6)	5469 (56.5)	10399 (58.1)	6373 (57.0)
	≥ 5 days/week	5838 (23.7)	2421 (25.0)	4024 (22.5)	2733 (24.4)
Seafood	Less frequently	2042 (8.3)	772 (8.0)	738 (4.1)	789 (7.1)
	1-2 days/week	12032 (48.8)	4879 (50.4)	8180 (45.7)	5507 (49.3)
	3-4 days/week	9099 (36.9)	3537 (36.6)	7606 (42.5)	4238 (37.9)
	≥ 5 days/week	1491 (6.0)	485 (5.0)	1360 (7.6)	644 (5.8)

© 2025 Suzuki T et al. *JAMA Network Open*.

		No alcohol-initiation cohort 24,664 participants	Alcohol-initiation cohort 9,673 participants	Alcohol-continued cohort 17,884 participants	Alcohol cessation cohort 11,178 participants
Soy	Less frequently	1839 (7.5)	666 (6.9)	984 (5.5)	800 (7.2)
	1-2 days/week	8767 (35.5)	3564 (36.8)	6926 (38.7)	4164 (37.3)
	3-4 days/week	8435 (34.2)	3359 (34.7)	6220 (34.8)	3871 (34.6)
	≥ 5 days/week	5623 (22.8)	2084 (21.5)	3754 (21.0)	2343 (21.0)
Milk and daily product	Less frequently	2197 (8.9)	932 (9.6)	1764 (9.9)	1055 (9.4)
	1-2 cup/week	3799 (15.4)	1568 (16.2)	3160 (17.7)	1894 (16.9)
	3-4 cup/week	4976 (20.2)	2021 (20.9)	3877 (21.7)	2287 (20.5)
	1 cup/day	10394 (42.1)	4027 (41.6)	7340 (41.0)	4679 (41.9)
	≥ 1.5 cups/day	3298 (13.4)	1125 (11.6)	1743 (9.7)	1263 (11.3)
Vegetables	Almost none	234 (0.9)	96 (1.0)	107 (0.6)	110 (1.0)
	Not everyday	1218 (4.9)	487 (5.0)	703 (3.9)	522 (4.7)
	1-2 dishes/day	10085 (40.9)	3999 (41.3)	7381 (41.3)	4856 (43.4)
	3-4 dishes/day	9457 (38.3)	3734 (38.6)	7229 (40.4)	4201 (37.6)
	5-6 dish/day	3148 (12.8)	1172 (12.1)	2144 (12.0)	1286 (11.5)
	≥ 7 dishes/day	522 (2.1)	185 (1.9)	320 (1.8)	203 (1.8)
Fruits	Less frequently	3730 (15.1)	1859 (19.2)	3693 (20.6)	2119 (19.0)
	1-2 dishes/week	4971 (20.2)	2114 (21.9)	3992 (22.3)	2465 (22.1)
	3-4 dishes/week	3880 (15.7)	1510 (15.6)	2887 (16.1)	1805 (16.1)
	1 dish/day	8718 (35.3)	3177 (32.8)	5785 (32.3)	3665 (32.8)
	2 dishes/day	2861 (11.6)	891 (9.2)	1346 (7.5)	984 (8.8)
	≥ 3 dishes/day	504 (2.0)	122 (1.3)	181 (1.0)	140 (1.3)
Sweets	Less frequently	2338 (9.5)	1064 (11.0)	3707 (20.7)	1383 (12.4)
	1-2 days/week	7075 (28.7)	3092 (32.0)	6362 (35.6)	3616 (32.3)
	3-4 days/week	7384 (29.9)	2878 (29.8)	4457 (24.9)	3299 (29.5)
	≥ 5 days/week	7867 (31.9)	2639 (27.3)	3358 (18.8)	2880 (25.8)
Fatty Diet	Almost none	5135 (20.8)	1713 (17.7)	3047 (17.0)	1904 (17.0)
	Sometimes	16308 (66.1)	6572 (67.9)	12464 (69.7)	7628 (68.2)
	Often	3221 (13.1)	1388 (14.3)	2373 (13.3)	1646 (14.7)

© 2025 Suzuki T et al. *JAMA Network Open*.

		No alcohol-initiation cohort 24,664 participants	Alcohol-initiation cohort 9,673 participants	Alcohol-continued cohort 17,884 participants	Alcohol cessation cohort 11,178 participants
Eating until becoming full	Almost none	3509 (14.2)	1339 (13.8)	2665 (14.9)	1462 (13.1)
	Sometimes	13970 (56.6)	5531 (57.2)	10795 (60.4)	6436 (57.6)
	Often	7185 (29.1)	2803 (29.0)	4424 (24.7)	3280 (29.3)
Eating out	Less frequently	11829 (48.0)	3924 (40.6)	5915 (33.1)	3714 (33.2)
	1-2 days/week	9213 (37.4)	4089 (42.3)	8068 (45.1)	5073 (45.4)
	3-4 days/week	2216 (9.0)	1028 (10.6)	2605 (14.6)	1546 (13.8)
	≥ 5 days/week	1406 (5.7)	632 (6.5)	1296 (7.2)	845 (7.6)
Snacks between meals	Almost none	4372 (17.7)	1942 (20.1)	6167 (34.5)	2457 (22.0)
	Sometimes	12899 (52.3)	5187 (53.6)	8683 (48.6)	6018 (53.8)
	Often	7393 (30.0)	2544 (26.3)	3034 (17.0)	2703 (24.2)

© 2025 Suzuki T et al. *JAMA Network Open*.

eTable 4. Multivariable adjusted models evaluating the association between initiating and ceasing alcohol consumption and changes in lipid concentration with 99% confidence intervals

**Alcohol Initiation (Cohort 1)**			
**Exposure**	Model 1, β (99% CI)	Model 2, β (99% CI)	Model 3, β (99% CI)
**LDL-C change**			
One unit increase of alcohol	-1.58 (-1.91 to -1.24)	-1.60 (-1.94 to -1.27)	-1.59 (-1.92 to -1.25)
Overall initiation	-1.26 (-1.68 to -0.84)	-1.29 (-1.70 to -0.87)	-1.27 (-1.69 to -0.85)
**HDL-C change**			
One unit increase of alcohol	1.14 (1.00 to 1.28)	1.15 (1.01 to 1.29)	1.15 (1.01 to 1.29)
Overall initiation	0.84 (0.66 to 1.02)	0.85 (0.68 to 1.03)	0.85 (0.68 to 1.03)
**Alcohol cessation (Cohort 2)**			
**LDL-C change**			
One unit decrease of alcohol	1.39 (1.11 to 1.67)	1.41 (1.13 to 1.69)	1.41 (1.13 to 1.69)
Overall cessation	1.41 (0.94 to 1.88)	1.48 (1.01 to 1.95)	1.48 (1.01 to 1.95)
**HDL-C change**			
One unit decrease of alcohol	-1.26 (-1.39 to -1.13)	-1.25 (-1.38 to -1.12)	-1.25 (-1.39 to -1.13)
Overall cessation	-1.62 (-1.84 to -1.41)	-1.59 (-1.80 to -1.37)	-1.59 (-1.80 to -1.37)

Model 1: Adjusted for age, sex, BMI, prior LDL-C/HDL-C cholesterol
Model 2: Adjusted for covariates in model 1 plus history of hypertension, history of diabetes, dietary habits (frequency of carbohydrates; meat and eggs; seafood; vegetables; fruits; milk and dairy products; soy; fat-rich diet; sweets, and frequency of eating until full, eating out and snacks between meals)
Model 3: Adjusted for covariates in model 2 plus smoking, daily physical activity and exercise level
In cohort 2, analyses additionally adjusted for the amount of alcohol intake and frequency of alcohol intake

© 2025 Suzuki T et al. *JAMA Network Open*.

eFigure 1. Subgroup analysis of adjusted association between initiating alcohol consumption (categorical) and changes in LDL cholesterol

Results from the fully-adjusted model (Model 3) are presented. LDL, low density lipoprotein; HDL, high density lipoprotein

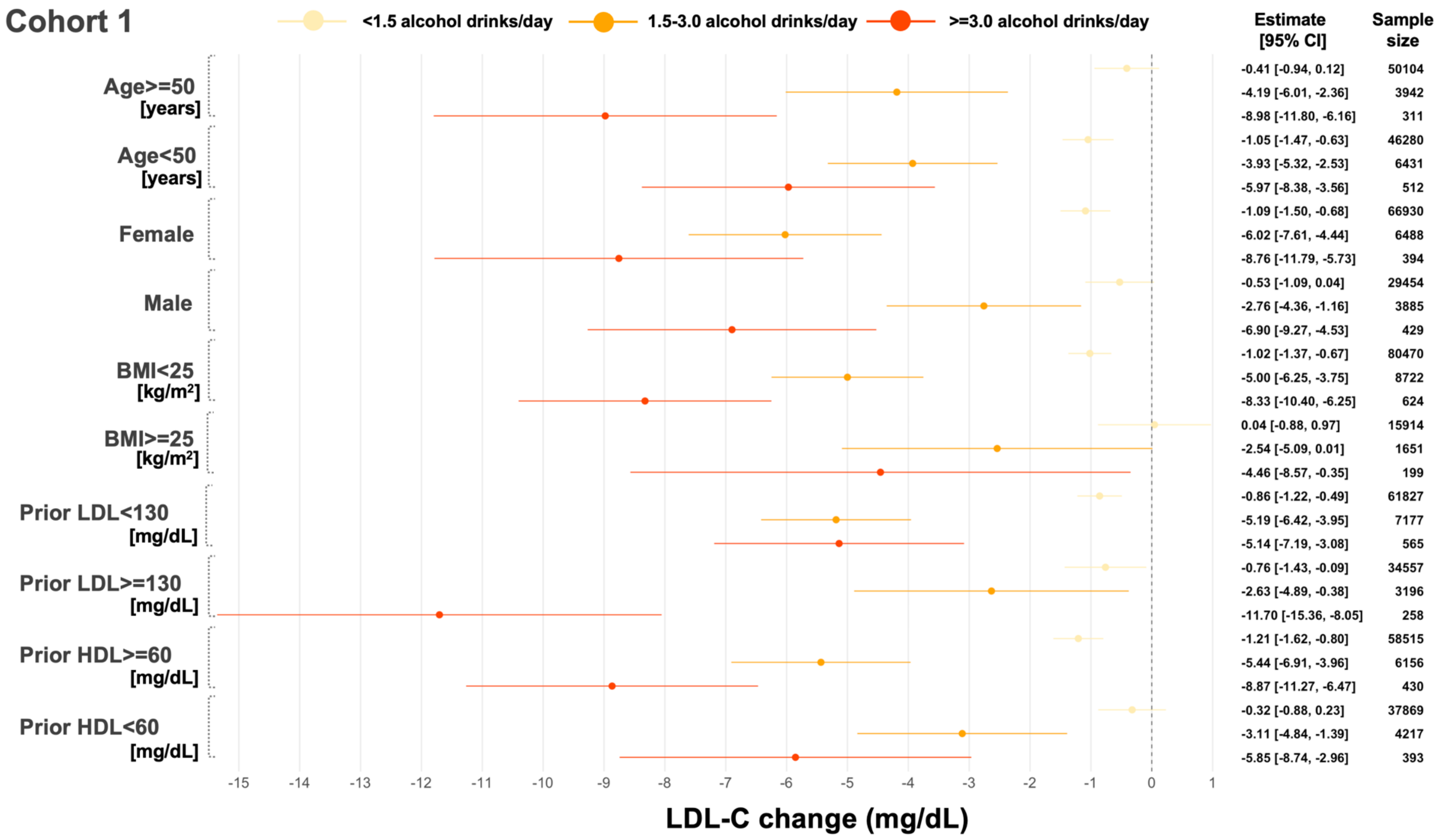

© 2025 Suzuki T et al. *JAMA Network Open*.

eFigure 2. Subgroup analysis of adjusted association between initiating alcohol consumption (categorical) and changes in HDL cholesterol
Results from the fully-adjusted model (Model 3) are presented. LDL, low density lipoprotein; HDL, high density lipoprotein

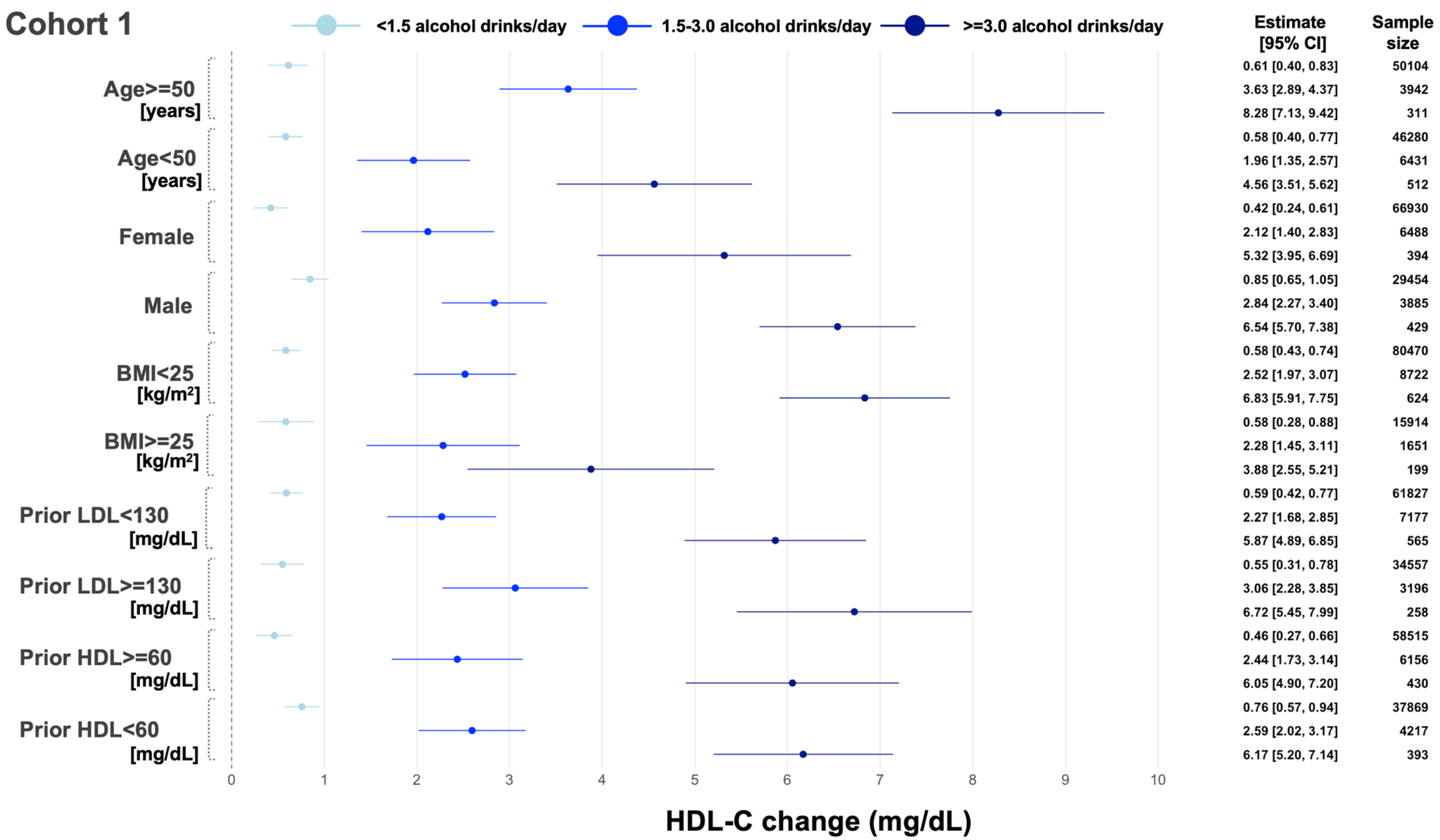

© 2025 Suzuki T et al. *JAMA Network Open*.

eFigure 3. Subgroup analysis of adjusted association between ceasing alcohol consumption (categorical) and changes in LDL cholesterol

Results from the fully-adjusted model (Model 3) are presented. LDL, low density lipoprotein; HDL, high density lipoprotein

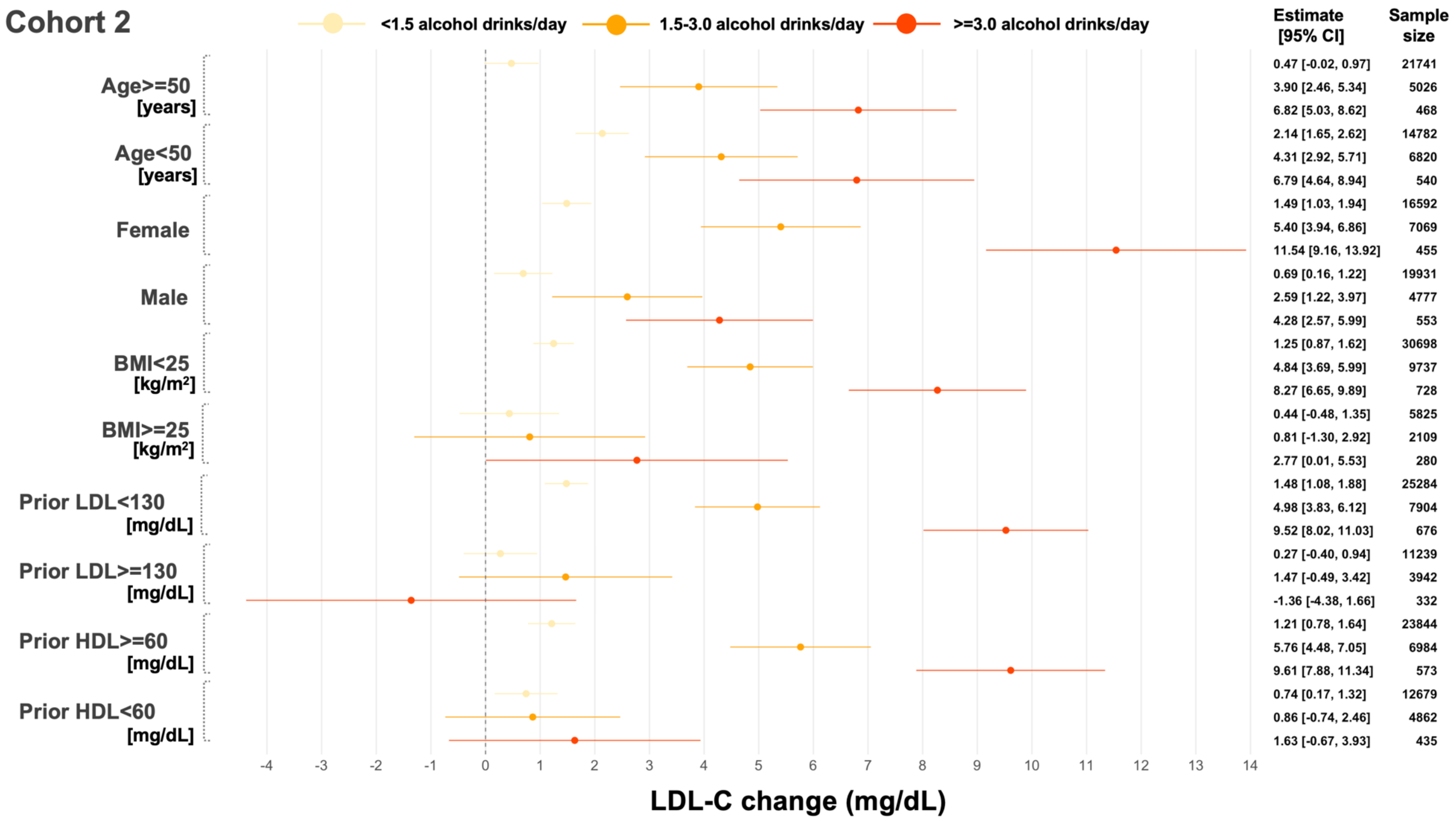

© 2025 Suzuki T et al. *JAMA Network Open*.

eFigure 4. Subgroup analysis of adjusted association between ceasing alcohol consumption (categorical) and changes in HDL cholesterol

Results from the fully-adjusted model (Model 3) are presented. LDL, low density lipoprotein; HDL, high density lipoprotein

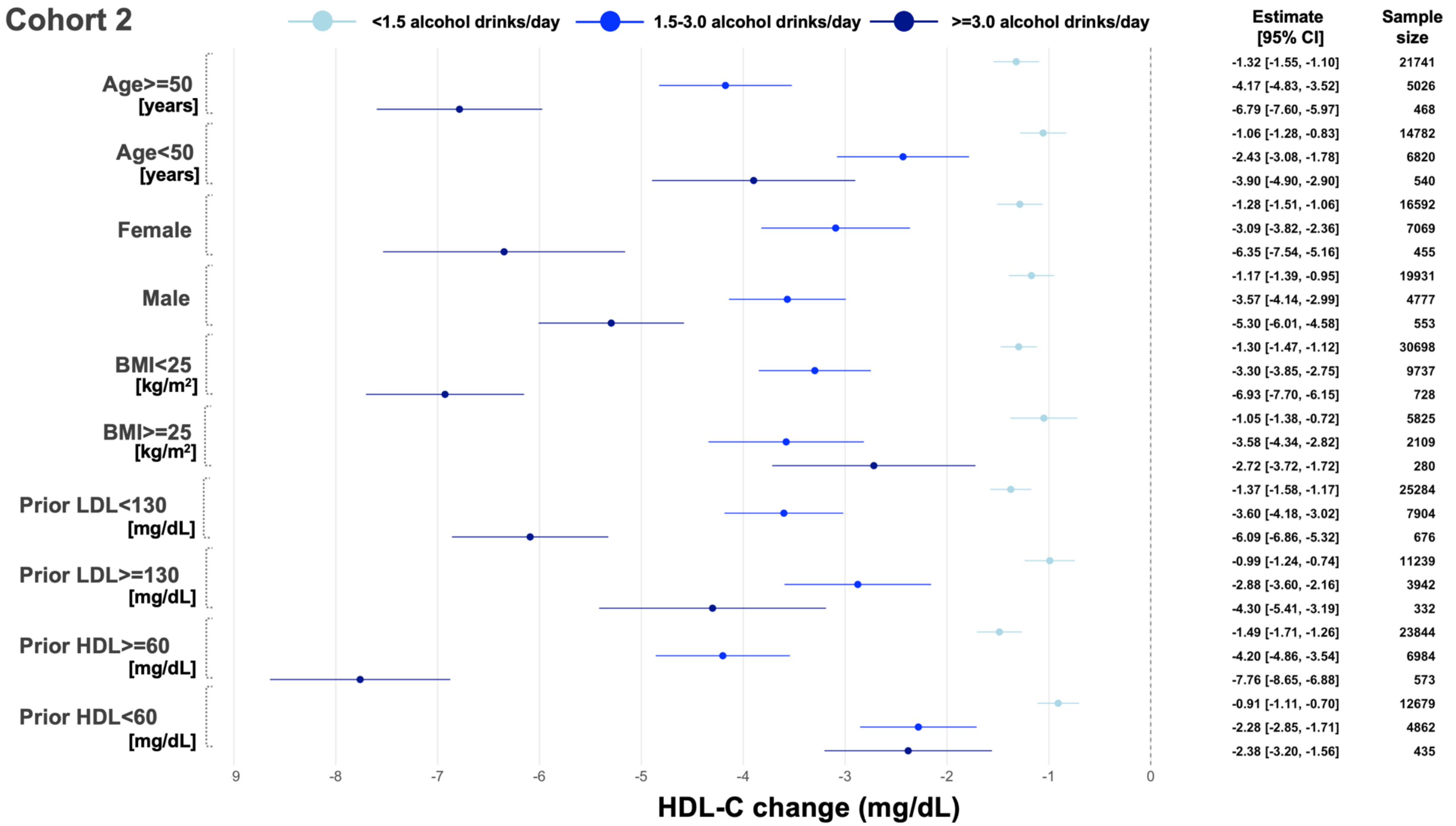

© 2025 Suzuki T et al. *JAMA Network Open*.
