# Supplementary material for: Lipid Profiles After Changes in Alcohol Consumption Among Adults Undergoing Annual Checkups
Source: JAMA Netw Open. 2025 Mar 12;8(3):e250583. doi: 10.1001/jamanetworkopen.2025.0583 (PMC11904732; doi:10.1001/jamanetworkopen.2025.0583)
Supplement: Supplement 2. — Data Sharing Statement [file jamanetwopen-e250583-s002.pdf]

Data Sharing Statement

Suzuki. Initiating and Ceasing Alcohol Consumption and Lipid Profile Changes in a Health Check-up Population. *JAMA Netw Open*. Published March 12, 2025.
doi:10.1001/jamanetworkopen.2025.0583

Data
Data available: No

Additional Information
Explanation for why data not available: Data is not publicly available due to the regulation in our institution. Data can be available on reasonable request from the corresponding author only after approval from the institution.
